# Supplementary material for: The role of 18F-FDG PET/CT in patients with diffuse large B-cell lymphoma after radioimmunotherapy using 131I-rituximab as consolidation therapy
Source: PLoS One. 2022 Sep 26;17(9):e0273839. doi: 10.1371/journal.pone.0273839 (PMC9512194; doi:10.1371/journal.pone.0273839)
Supplement: Attachment — Submitted filename: Response to Reviewers.docx [file pone.0273839.s002.docx]

We would like to resubmit this manuscript [PONE-D-22-14199] - [EMID:29b7a70172fff9e1], entitled “The role of 18F-FDG PET/CT in patients with diffuse large B-cell lymphoma after radioimmunotherapy using 131I-rituximab as consolidation therapy” to *PLOS ONE* for publication. We appreciate the careful reviews provided by the editor and reviewers. We are submitting a revised version of our manuscript in which we have considered or responded to all of the comments and questions. Our detailed responses are given below. We have also replied to the reviewers’ comments in a point-by-point manner, and the changes made have been highlighted in red in the revised manuscript.

**1. Reviewer #1**

**(Note: Blue color highlights the text that was deleted and red color highlights the text that was added.)**

**Reviewer’s comment #1 (minor)**

1. page 14, line 124-125: potassium ioidide was also provided at least 24 hr~. I think the authors should add "for thyroid protection", as some readers may not be familiar with I-131 rituximab treatment protocol.

**Author’s response for reviewer’s comment #1**

: Thank you for your comment; we modified the sentence.

**Text changes for reviewer’s comment #1**

**Materials and Methods section, Treatment protocols paragraph**

Potassium iodide was also provided at least 24 h prior to 131I-rituximab administration.

🡪

Potassium iodide was also provided at least 24 h prior to 131I-rituximab administration for thyroid protection.

**Reviewer’s comment #2 (minor)**

2. I am not familiar with I-131 rituximab being administered as consolidation therapy. Although it is not possible to compare with other patients who achieved complete remission and did not recieve I-131 rituximab, I think the authors should add a short discription on the possible advantages or reported advantages of this treatment protocol.

**Author’s response for reviewer’s comment #2**

Thank you for your comment. We totally agreed with your opinion. We added the rationale of consolidation therapy in lymphoma patient. We also modified the paragraph to focus on integrative therapy to make the protocol easier for readers to understand. We also introduced the clinical efficacy of previous phase 2 studies.

**Text changes for reviewer’s comment #2**

**Introduction section**

Diffuse large B-cell lymphoma (DLBCL) is characterized by an aggressive phenotype. It is the most frequent type of non-Hodgkin lymphoma (NHL), accounting for 30–40% of all NHL cases [1, 2]. Over the last decade, the addition of rituximab to traditional cyclophosphamide, doxorubicin, vincristine, and prednisone (R-CHOP) chemotherapy has led to significant advances and improvements of treatment response and survival for patients with DLBCL [3, 4]. Despite therapeutic advances with the R-CHOP regimen, about 40% of patients with DLBCL cannot be completely cured with R-CHOP therapy.

Radioimunotherapy (RIT) is a treatment option that uses Ytrtrium-90 or Iodine-131 conjugated to anti-CD20 monoclonal antibodies to release high-energy, short-path distance radiation to tumor cells while avoiding damage to adjacent normal tissues. Based on treatment outcomes in studies on relapsed indolent NHL, the US FDA have approved 90Y-ibritumomab tiuxetan (Zevalin, Spectrum pharmaceuticals) for relapsed low grade, follicular B-cell NHL [5-7]. 131I-tositumomab (Bexxar, GlaxoSmithKline) has also been approved for relapsed low grade, follicular, and transformed NHL [8]. We have given a single dose or a repeated dose of 131I-rituximab to patients with relapsed/refractory CD20 positive B cell lymphoma followed by a consolidation therapy after 6-8 cycles of R-CHOP for those who have achieved complete remission [9-11].

🡪

Diffuse large B-cell lymphoma (DLBCL) is characterized by an aggressive phenotype. It is the most frequent type of non-Hodgkin lymphoma (NHL), accounting for 30–40% of all NHL cases [1, 2]. Over the last decade, the addition of rituximab to traditional cyclophosphamide, doxorubicin, vincristine, and prednisone (R-CHOP) chemotherapy has led to significant advances and improvements of treatment response and survival for patients with DLBCL [3, 4]. Despite therapeutic advances with the R-CHOP regimen, about 40% of patients with DLBCL cannot be completely cured with R-CHOP therapy. Owing to the high risk of treatment failure, many researchers have suggested consolidation therapy such as autologous stem cell transplantation (ASCT) [5]. However, many patients have experienced difficulties on receiving ASCT due to the advanced age or poor performance.

Radioimunotherapy (RIT) is a treatment option that uses Ytrtrium-90 or Iodine-131 conjugated to anti-CD20 monoclonal antibodies to release high-energy, short-path distance radiation to tumor cells while avoiding damage to adjacent normal tissues. ^90^Y-ibritumomab (Zevalin, Spectrum Pharmaceuticals) is the only RIT currently approved in Europe, and its successful use in consolidation treatment following chemotherapy has been well documented in patients with follicular lymphoma (FL), and chronic lymphocytic leukemia (CLL) [6, 7]. The use of ^131^I-tositumomab (Bexxar, GlaxoSmithKline) as a consolidation therapy has also been reported in CLL, advanced DLBCL, and FL as a consolidation therapy [8-10]. Meanwhile, a study using rituximab as a consolidation therapy was conducted in a phase 2 clinical study at our institution [11]. This study is based on the rationale that RIT using ^131^I and Zevalin have the same conditions for targeting CD20 positive B-Cell lymphoma, but due to different physical and biological properties, a difference in survival rate was expected. In the phase 2 trial study, ^131^I-rituximab showed promising efficacy as consolidation treatment for patients with DLBCL.

**2. Reviewer #2**

**(Note: Blue color highlights the text that was deleted and red color highlights the text that was added.)**

**Reviewer’s comment #1**

1. The details of the study are not sufficient. Was this a phase I study? What was the rationale for the study? Consolidation for DLBCL is not standard of care? How were patients selected and why? What were the intended benefits? What ethical approval was obtained and what is the clinical trial number? More details on trials/proposed consolidation therapy need to be included.

**Author’s response for reviewer’s comment #1**

: Thank you for your comment. We totally agree that the description of the method section is insufficient and supplement was added.

Firstly, this study is a retrospective review of a prospective phase II clinical trial. This study is based on the rationale that radioimmunotherapy using I131 and Zevalin have the same conditions for targeting CD20 positive B-Cell lymphoma, but due to different physical properties (PMID: 27067042, PMID: 16550338), a difference in survival rate was expected. In the prospective, phase II study, I131-rituximab showed promising efficacy as consolidation treatment for patients with DLBCL. This clinical trial was conducted as an investigator-led, open-label, non-randomized, single-center trial conducted by the Korea Cancer Center in Republic of Korea in 2004. This study was expected the improvement of progression free survival in patients achieving complete response through standard chemotherapy using I131-rituximab. The final protocol was approved by the institutional review board and registered with the World Health Organization - International Clinical Trials Registration Platform (WHO-ICTRP ID: KCT0000526).

**Text changes for reviewer’s comment #1**

**Introduction section**

Diffuse large B-cell lymphoma (DLBCL) is characterized by an aggressive phenotype. It is the most frequent type of non-Hodgkin lymphoma (NHL), accounting for 30–40% of all NHL cases [1, 2]. Over the last decade, the addition of rituximab to traditional cyclophosphamide, doxorubicin, vincristine, and prednisone (R-CHOP) chemotherapy has led to significant advances and improvements of treatment response and survival for patients with DLBCL [3, 4]. Despite therapeutic advances with the R-CHOP regimen, about 40% of patients with DLBCL cannot be completely cured with R-CHOP therapy.

Radioimunotherapy (RIT) is a treatment option that uses Ytrtrium-90 or Iodine-131 conjugated to anti-CD20 monoclonal antibodies to release high-energy, short-path distance radiation to tumor cells while avoiding damage to adjacent normal tissues. Based on treatment outcomes in studies on relapsed indolent NHL, the US FDA have approved 90Y-ibritumomab tiuxetan (Zevalin, Spectrum pharmaceuticals) for relapsed low grade, follicular B-cell NHL [5-7]. 131I-tositumomab (Bexxar, GlaxoSmithKline) has also been approved for relapsed low grade, follicular, and transformed NHL [8]. We have given a single dose or a repeated dose of 131I-rituximab to patients with relapsed/refractory CD20 positive B cell lymphoma followed by a consolidation therapy after 6-8 cycles of R-CHOP for those who have achieved complete remission [9-11].

🡪

Diffuse large B-cell lymphoma (DLBCL) is characterized by an aggressive phenotype. It is the most frequent type of non-Hodgkin lymphoma (NHL), accounting for 30–40% of all NHL cases [1, 2]. Over the last decade, the addition of rituximab to traditional cyclophosphamide, doxorubicin, vincristine, and prednisone (R-CHOP) chemotherapy has led to significant advances and improvements of treatment response and survival for patients with DLBCL [3, 4]. Despite therapeutic advances with the R-CHOP regimen, about 40% of patients with DLBCL cannot be completely cured with R-CHOP therapy. Owing to the high risk of treatment failure, many researchers have suggested consolidation therapy such as autologous stem cell transplantation (ASCT) [5]. However, many patients have experienced difficulties on receiving ASCT due to the advanced age or poor performance.

Radioimunotherapy (RIT) is a treatment option that uses Ytrtrium-90 or Iodine-131 conjugated to anti-CD20 monoclonal antibodies to release high-energy, short-path distance radiation to tumor cells while avoiding damage to adjacent normal tissues. ^90^Y-ibritumomab (Zevalin, Spectrum Pharmaceuticals) is the only RIT currently approved in Europe, and its successful use in consolidation treatment following chemotherapy has been well documented in patients with follicular lymphoma (FL), and chronic lymphocytic leukemia (CLL) [6, 7]. The use of ^131^I-tositumomab (Bexxar, GlaxoSmithKline) as a consolidation therapy has also been reported in CLL, advanced DLBCL, and FL as a consolidation therapy [8-10]. Meanwhile, a study using rituximab as a consolidation therapy was conducted in a phase 2 clinical study at our institution [11]. This study is based on the rationale that RIT using ^131^I and Zevalin have the same conditions for targeting CD20 positive B-Cell lymphoma, but due to different physical and biological properties, a difference in survival rate was expected. In the phase 2 trial study, ^131^I-rituximab showed promising efficacy as consolidation treatment for patients with DLBCL.

**Materials and Methods section, Patient enrollment paragraph**

We retrospectively reviewed a prospective study initiated in December 2004 and conducted at the Korea Caner Center Hospital in the Republic of Korea [9].

🡪

We retrospectively reviewed a prospective, single-center phase II trial study initiated in December 2004 and conducted at the Korea Caner Center Hospital in the Republic of Korea (WHO-ICTRP ID: KCT0000526) [10].

**Reviewer’s comment #2**

2. In the treatment outcomes and follow up section it mentions three patients died during follow up? What did they did of? Progressive disease or another reason? (line 186)

**Author’s response for reviewer’s comment #2**

: Thank you for your comment. All of them had died of disease progression. We have rewritten the sentence with the above content.

**Text changes for reviewer’s comment #2**

Among 15 patients who received consolidative 131I-rituximab, six achieved complete response (CR) after three cycles of R-CHOP and 9 achieved CR after six or eight cycles of R-CHOP. All fifteen patients received consolidation RIT after complete response. The median time from the day of the last R-CHOP administration to RIT was 48 days (range, 32–59 days). The median follow-up period of patients was 73 months (range, 11-108 months). Four (27%) patients relapsed. Of them, three died during follow up.

🡪

Among 15 patients who received consolidative 131I-rituximab, six achieved complete response (CR) after three cycles of R-CHOP and 9 achieved CR after six or eight cycles of R-CHOP. All fifteen patients received consolidation RIT after complete response. The median time from the day of the last R-CHOP administration to RIT was 48 days (range, 32–59 days). The median follow-up period of patients was 73 months (range, 11-108 months). Four (27%) patients relapsed. Of them, three died during follow up. In one patient, follow-up magnetic resonance imaging showed brain metastasis 2 months after the administration of ^131^I-rituximab, and the patient died 9 months later due to disease progression. Another patient, who received ^131^I-rituximab, had a relapse of the disease 31 months later, and survived for 34 months before he died of disease progression. The last patient survived for 43 months after treatment.

**Reviewer’s comment #3**

3. Two case descriptions are given (lines 259-291). Whilst interesting this is not a standard way to report outcomes in clinical trial. A swimmers plot including all patients enrolled with details about outcomes (relapse/death etc) would be more appropriate.

**Author’s response for reviewer’s comment #3**

: Thank you for your comment. We totally agree with your comments. We have created figure 1 as a swimmer’s plot with survival details of 15 patients.

**Text changes for reviewer’s comment #3**

**Result section, Treatment outcomes and follow-up results paragraph**

Fig 1. A swimmer plot showing the survival period of 15 people. Four people relapsed, of whoem three expired. Those who relapsed are indicated by a red triangle, and those who survived are indicated by a black arrow.

**Reviewer’s comment #4**

4. I do not agree with the conclusions or they are not explained adequately. The authors conclude that PET/CT pretreatment can be used to predict survival in DLBCL in patients receiving consolidation therapy. Does pretreatment PET/CT not predict outcomes for all patients regardless of whether they received consolidation therapy or not? It would be useful to know the impact of SUVmax pretreatment on survival outcomes of those who don't receive consolidation therapy (either from the literature or comparator cohort from the same institution). The authors conclude that the sex of the patient is associated with prognosis yet there are only 3 females in the cohort! These numbers and the skewed sex ratio of the cohort make it impossible to make any associations between sex and outcomes.

**Author’s response for reviewer’s comment #4**

: Thank you for your comments. We totally agree with reviewer’s opinion. In consolidation therapy using I-131 rituxmab in DLBCL patients, there are not many studies investigating the prognostic factors of PET/CT. Thus, among DLBCL patients who did not undergo consolidation, studies in which SUVmax as a prognostic factor were compared with ours, and this was added to the discussion section.

In addition, sentences about sex ratios that are inappropriate for statistical analysis were deleted.

**Text changes for reviewer’s comment #4**

**Discussion section, Third paragraph (added)**

In our study, it was found that a high SUVmax showed better treatment effect in those who received consolidation therapy using RIT for DLBCL patients. Although, there are not many studies examining the prognostic factor of PET/CT in RIT consolidation therapy after conventional chemotherapy in DLBCL patients, we compared our results with a study that investigated prognostic factors in patients who had not undergone consolidation therapy. In one meta-analysis, SUVmax and MTV were significant prognostic factors for progression free survival (PFS), but only MTV was reported as a significant factor for OS [35]. Also, in a review study of 20 studies, SUVmax showed significant predictive ability for PFS and OS in 5 studies compared to 17 out of 19 for MTV and 10 out of 13 for TLG showing significant predictive ability for both PFS and OS [36]. A recent study reported that SUVmax was either undetermined or was a prognostic factor only in PFS [37, 38].

**Reviewer’s comment #5**

5. The limitations section needs to be significantly expanded and rewirtten. The context of the study is not clear and the list of limitations is long.

Overall the standard of English needs to be significantly improved, particularly the discussion section.

**Author’s response for reviewer’s comment #5**

: Thank you for your comment. We totally agree with reviewer’s opinion. We have rewritten the limitation paragraph and specifically expanded the paragraphs that come from the study design. We also corrected awkward sentences and grammatical errors in the discussion, and received professional English proofreading.

**Text changes for reviewer’s comment #5**

**Discussion section, fifth paragraph (modified)**

Regarding the mechanism of prediction, Gallicchio et al. have demonstrated better survival for patients with DLBCL having higher values of SUVmax who underwent 18F-FDG PET/CT imaging before receiving chemotherapy [24] (p = 0.0002, HR: 0.13, 95% CI: 0.04-0.46). The present study showed similar results, which was expected because higher level of glycolytic activity was associated with better response to chemotherapy. Furthermore, patients with large decline of SUVmax in post-treatment PET also have good prognosis in some types of carcinoma [34-36]. This suggests that higher SUV in pretreatment PET may predict better prognosis with good response to treatment. The prognostic value of 18F-FDG PET/CT in lymphoma has been established [37]. Meanwhile, the uptake of 18F-FDG PET/CT in lymphoma is influenced diversely by multifactorial process of various biological factors such as oncogene expression, apoptosis and viable cell fraction, impacts of hypoxia, extent of inflammatory cell infiltration, and tumor location (nodal/extranodal) [38]. Therefore, uptake heterogeneity could be encountered in patients with the same histologic subtype [37].

🡪

Regarding the mechanism of prediction, Gallicchio et al. have demonstrated better survival for patients with DLBCL having higher values of SUVmax who underwent ^18^F-FDG PET/CT imaging before receiving chemotherapy [25] (*p* = 0.0002, HR: 0.13, 95% CI: 0.04-0.46). The present study showed similar results, which was expected because higher level of glycolytic activity was associated with better response to chemotherapy. Furthermore, patients with large decline of SUVmax in post-treatment PET also have good prognosis in some types of carcinoma [39-41]. This suggests that higher SUV in pretreatment PET may predict better prognosis with good response to treatment. The prognostic value of ^18^F-FDG PET/CT in lymphoma has been established [42]. The uptake of ^18^F-FDG PET/CT in lymphoma is influenced diversely by multifactorial process of various biological factors such as oncogene expression, apoptosis and viable cell fraction, impacts of hypoxia, extent of inflammatory cell infiltration, and tumor location (nodal/extranodal) [43]. This can lead to uptake heterogeneity in patients with the same histologic subtype [42].

**Discussion section, sixth paragraph (modified)**

Another some mechanisms can explain our results, low SUVs might be associated with hypoxic tumors that can be less aggressive if not treated, but paradoxically more resistant to chemotherapy treatment [39]. Other possible mechanisms might be because those with higher cell proliferation have higher SUV values [40]. Thus, they are more likely to respond to treatment [34, 41]. *In vitro* studies have revealed that cells resistant to chemotherapy show decreased FDG uptake, suggesting that altered glucose transporter may transport into resistant tumor cells [42, 43].

🡪

There are some mechanisms that can explain our results. Low SUVs could be associated with hypoxic tumors that can be less aggressive if not treated, but paradoxically more resistant to chemotherapy treatment [44]. Another possible explanation is that those with higher cell proliferation have higher SUV values [45], and those are more likely to respond to treatment [39, 46]. *In vitro* studies have revealed that cells resistant to chemotherapy show decreased FDG uptake, suggesting that altered glucose transporter may transport into resistant tumor cells [47, 48].

**Discussion section, eight paragraph (modified)**

The present study has the following limitations. First, a small number of patients were recruited. This was because the study was terminated due to poor enrollment. Second, only pretreatment images were obtained. Response of the tumor could not be evaluated because only patients with CR were enrolled. The present study revealed that higher SUVmax and larger tumor size were associated with good prognosis in RFS. It also revealed that higher SUVmax and male patient were associated with good prognosis in OS. Although there are several limitations present in this study, it is concerned that 131I-rituximab may not respond to consolidation therapy in pretreatment DLBCL patients with low SUVmax and female patient. Further studies are needed to improve the prognostic performance of RIT consolidation therapy in DLBCL patients for clinical treatment decisions.

In conclusion, Higher SUVmax at pretreatment 18F-FDG PET/CT demonstrated better relapse free survival and overall survival in DLBCL patients after a consolidation therapy of 131I-rituximab. Pretreatment 18F-FDG PET/CT can be used for predicting survival in DLBCL patients after a consolidation therapy of 131I-rituximab.

🡪

The present study has the following limitations. First, only small number of patients were recruited due to low enrollment. This was because the study had a difficulty with enrolling patients for consolidation therapy after complete remission had occurred. Second, non-randomization of the study limits the interpretation of its results. Only patients who received RIT consolidation therapy were included and not the ones who only received chemotherapy. Third, only pretreatment images were obtained. Tumor response could not be evaluated because only patients with CR were enrolled. Fourth, this study has limitations as it is a retrospective study, though data were collected prospectively. Despite these limitations, we consider it important to report our findings in the article in view of the prognostic value of 18F-FDG PET/CT in RIT consolidation therapy for DLBCL patients.

In conclusion, our result showed higher SUVmax at pretreatment 18F-FDG PET/CT was associated with better relapse free survival and overall survival in DLBCL patients who received consolidation therapy with 131I-rituximab. However, since the study was conducted with a small number of subjects and was limited to consolidation therapy patients, a phase 3 trial studies with a larger number of patients are needed before clinical application.
