# Supplementary material for: The role of 18F-FDG PET/CT in patients with diffuse large B-cell lymphoma after radioimmunotherapy using 131I-rituximab as consolidation therapy
Source: PLoS One. 2022 Sep 26;17(9):e0273839. doi: 10.1371/journal.pone.0273839 (PMC9512194; doi:10.1371/journal.pone.0273839)
Supplement: Attachment — Submitted filename: Response to Reviewers.docx [file pone.0273839.s003.docx]

We would like to resubmit this manuscript [PONE-D-22-14199R1] - [EMID:3c112168cfebaa1e], entitled “The role of 18F-FDG PET/CT in patients with diffuse large B-cell lymphoma after radioimmunotherapy using 131I-rituximab as consolidation therapy” to *PLOS ONE* for publication. We appreciate the careful reviews provided by the editor and reviewers. We are submitting a revised version of our manuscript in which we have considered or responded to all of the comments and questions. Our detailed responses are given below. We have also replied to the reviewers’ comments in a point-by-point manner, and the changes made have been highlighted in red in the revised manuscript.

**1. Reviewer #2**

**(Note: Blue color highlights the text that was deleted and red color highlights the text that was added.)**

**Reviewer’s comment #4**

4. Have the authors made all data underlying the findings in their manuscript fully available?

Reviewer #2: No

**Author’s response for reviewer’s comment #4**

: We have already attached anonymized files of patients in the last revision.

**Text changes for reviewer’s comment #4**

No change

**Reviewer’s comment #5**

5. Is the manuscript presented in an intelligible fashion and written in standard English?

Reviewer #2: No

**Author’s response for reviewer’s comment #5**

: Thank you for your comments. We have requested the correction of the latest English text and grammatical errors and typos from the English proofreading company. The certificate of editing is also attached.

**Text changes for reviewer’s comment #5**

1) **Introduction section, second paragraph**

Radioimunotherapy (RIT) is a treatment option that uses Ytrtrium-90 or Iodine-131 conjugated to anti-CD20 monoclonal antibodies to release high-energy, short-path distance radiation to tumor cells while avoiding damage to adjacent normal tissues. 90Y-ibritumomab (Zevalin, Spectrum Pharmaceuticals) is the only RIT currently approved in Europe, and its successful use in consolidation treatment following chemotherapy has been well documented in patients with follicular lymphoma (FL), and chronic lymphocytic leukemia (CLL) [6, 7]. The use of 131I-tositumomab (Bexxar, GlaxoSmithKline) as a consolidation therapy has also been reported in CLL, advanced DLBCL, and FL as a consolidation therapy [8-10]. Meanwhile, a study using rituximab as a consolidation therapy was conducted in a phase 2 clinical study at our institution [11]. This study is based on the rationale that RIT using 131I and Zevalin have the same conditions for targeting CD20 positive B-Cell lymphoma, but due to different physical and biological properties, a difference in survival rate was expected. In the phase 2 trial study, 131I-rituximab showed promising efficacy as consolidation treatment for patients with DLBCL.

🡪

Radioimunotherapy (RIT) is a treatment option that uses Ytrtrium-90 or Iodine-131 conjugated to anti-CD20 monoclonal antibodies to release high-energy, short distance radiation to tumor cells while avoiding damage to adjacent normal tissues. 90Y-ibritumomab (Zevalin, Spectrum Pharmaceuticals) is the only RIT currently approved in Europe, and its successful use in consolidation treatment following chemotherapy has been well documented in patients with follicular lymphoma (FL), and chronic lymphocytic leukemia (CLL) [6, 7]. The use of 131I-tositumomab (Bexxar, GlaxoSmithKline) as a consolidation therapy has also been reported in CLL, advanced DLBCL, and FL [8-10]. Meanwhile, a study using rituximab as a consolidation therapy was conducted in a phase 2 clinical study at our institution [11]. This study is based on the rationale that RIT using 131I and Zevalin have a similar mechanism for targeting CD20 positive B-Cell lymphoma, but due to different physical and biological properties, a difference in survival rate was expected. In the phase 2 trial study, 131I-rituximab showed promising efficacy as consolidation treatment for patients with DLBCL.

2) **Materials and Methods section, Imaging analysis paragraph**

Standardized uptake value (SUV) was defined as the tissue concentration (MBq/mL) of the tracer divided by the amount injected per body weight (MBq/g). MIMvista workstation software version 6.8.3 (MIM Software Inc., Cleveland, OH, USA) was used for measuring the maximum standardized uptake values (SUVmax), metabolic tumor volume (MTV), and total lesion glycolysis (TLG) of lesions. Patient-based analysis was based on the highest SUVmax when multiple lesions were present. MTV was automatically obtained from the tumor volume combined with FDG uptake with a gradient-based method (PET edge) [15]. TLG was defined as MTV multiplied by SUVmean. The gradient method depends on the starting point near the center of the lesion used by an operator. When the operator decided the center of the lesion, six axes spread out for the starting point of gradient segmentation. Spatial gradients were assessed along each axis. If a large spatial gradient was discovered, the length of an axis was limited [2, 16, 17].

🡪

Standardized uptake value (SUV) was defined as the tissue concentration (MBq/mL) of the tracer divided by the amount injected per body weight (MBq/g). MIMvista workstation software version 6.8.3 (MIM Software Inc., Cleveland, OH, USA) was used for measuring the SUVmax, MTV, and TLG of lesions. Patient-based analysis was based on the highest SUVmax when multiple lesions were present. MTV was automatically obtained from the tumor volume combined with FDG uptake with a gradient-based method (PET edge) [15]. TLG was defined as MTV multiplied by SUVmean. The gradient method depends on the starting point at the center of the lesion determined by the operator. When the operator drags out from the lesion center, six axes spread out, providing visual feedback for the stating point. Spatial gradients were assessed along each axis. If a large spatial gradient was discovered, the length of an axis was limited [2, 16, 17].

3) **Results section, Imaging findings paragraph**

All patients underwent baseline image of 18F-FDG PET/CT before receiving R-CHOP treatment. A total of 50 lymph nodes of Ann Arbor classification in 15 patients showed positive FDG findings suggestive of lymphoma. According to the location of the lesion, there were 10 cases of cervical, supraclavicular lymphadenopathy, 9 cases of Waldeyer's ring, 7 cases of axillary lymph node, 7 cases of iliac lymph node, 5 cases of aortic lymph node, 4 cases of mediastinal lymph node, and 2 cases of brachial, mesenteric, inguinal lymph node and spleen, respectively.

The median value of largest tumor size was 5.3 cm (range, 2.0 – 16.4 cm). The largest lesions of patients were composed of 4 in supraclavicular LN, 3 in axillary LN, two in each case of neck and mesentery, one in each case of abdomen LN, liver, L spine, and sigmoid colon, respectively. The median value of the highest SUVmax was 20.2 (range, 11.1 - 67.4). Lesion sites with the highest SUVmax were found in five cases of cervical LN, 2 cases at abdominal LN and supraclavicular LN, and one case of common iliac LN, axillary LN, uterus, nasal cavity, tonsil, and sternum, respectively. MTV and TLG were also measured with a gradient-based PET segmentation method (PET edge). Median values of MTV and TLG were 231.51 (range, 15 – 38.34), 1277.95 (range, 238.37 – 10341.04), respectively.

🡪

All patients underwent baseline 18F-FDG PET/CT before receiving R-CHOP treatment. A total of 50 lymph nodes of Ann Arbor classification in 15 patients showed positive FDG findings suggestive of lymphoma. The location was as follows: 10 in cervical and supraclavicular LN; 9 in Waldeyer’s ring; 7 in axillary and iliac LN each; 5 in aortic LN; 4 in mediastinal LN; 2 in brachial, mesenteric, inguinal LN and spleen each.

The median largest tumor size was 5.3 cm (range, 2.0 – 16.4 cm). The distribution of the location of the largest tumor was as follows: supraclavicular LN, 4 patients; axillary LN, 3; neck and mesentery, 2 each; abdomen LN, liver, L-spine and sigmoid colon, 1 each. The median highest SUVmax was 20.2 (range, 11.1 - 67.4). The distribution of the location of the highest SUVmax was as follows: cervical LN, 5 patients; abdominal LN and supraclavicular LN, 2 each; common iliac LN, axillary LN, uterus, nasal cavity, tonsil and sternum, 1 each. MTV and TLG were also measured with a gradient-based PET segmentation method (PET edge). Median values of MTV and TLG were 231.51 (range, 15 – 38.34) and 1277.95 (range, 238.37 – 10341.04), respectively.

4) **Discussion section, 3rd paragraph**

In our study, it was found that a high SUVmax showed better treatment effect in those who received consolidation therapy using RIT for DLBCL patients. Although, there are not many studies examining the prognostic factor of PET/CT in RIT consolidation therapy after conventional chemotherapy in DLBCL patients, we compared our results with a study that investigated prognostic factors in patients who had not undergone consolidation therapy.

🡪

In our study, it was found that a high SUVmax showed better treatment effect in DLBCL patients who received RIT consolidation therapy. Although, there are not many studies examining the prognostic factors of PET/CT in DLBCL patients who received RIT consolidation therapy after conventional chemotherapy, we compared our results with a study that investigated prognostic factors in patients who had not undergone consolidation therapy.

5) Certificate of Editing

**Reviewer’s comment #6**

The authors have made substantial improvement to the manuscript. However I still have some major concerns. These mainly relate to how the study was conducted. In particular the sentence that states 'the requirement for informed consent was waived'. I cannot understand how a prospective trial of an investigational medicinal product could be conducted without informed consent from the participants. This goes against the Declaration of Helsinki and needs to be clarified before this manuscript can be considered for publication. In addition the standard of English is still poor and needs to be improved.

**Author’s response for reviewer’s comment #6**

: Thank you for your comments. Because this is a retrospective analysis of a previous prospective study, our Institutional Review Board allowed the waiver of informed consent. We have additionally modified the sentence to make this part clearer.

**Text changes for reviewer’s comment #6**

**Materials and Methods section, Patient enrollment paragraph**

Protocols of this study were approved by our Institutional Review Board (IRB No.: 2019-02-009), and the requirement to obtain informed consent was waived.

Protocols of this study were approved by our Institutional Review Board (IRB No.: 2019-02-009), and the requirement to obtain informed consent was waived in this retrospective analysis.
