# Supplementary material for: The role of 18F-FDG PET/CT in patients with diffuse large B-cell lymphoma after radioimmunotherapy using 131I-rituximab as consolidation therapy
Source: PLoS One. 2022 Sep 26;17(9):e0273839. doi: 10.1371/journal.pone.0273839 (PMC9512194; doi:10.1371/journal.pone.0273839)
Supplement: Attachment — Submitted filename: Response to Reviewers.docx [file pone.0273839.s004.docx]

We would like to resubmit this manuscript [PONE-D-22-14199R2] - [EMID:bb003148bf32d220], entitled “The role of 18F-FDG PET/CT in patients with diffuse large B-cell lymphoma after radioimmunotherapy using 131I-rituximab as consolidation therapy” to *PLOS ONE* for publication. We appreciate the careful reviews provided by the editor and reviewers. We are submitting a revised version of our manuscript in which we have considered or responded to all of the comments and questions. Our detailed responses are given below. We have also replied to the reviewers’ comments in a point-by-point manner, and the changes made have been highlighted in red in the revised manuscript.

**1. Reviewer #2**

**(Note: Blue color highlights the text that was deleted and red color highlights the text that was added.)**

**Reviewer’s comment #6**

Reviewer #2: The manuscript has been substantially improved and the proofreading has improved the quality of the manuscript. I appreciate that consent may have been waived for the retrospective analysis in this manuscript but the authors still need to detail the consent process, ethical approvals for use of data in this way in order to make the manuscript suitable for publication.

**Author’s response to reviewer’s comment #4**

: Thank you for your comments. The Institutional Review Board allows exceptions to be exempted from the informed consent of the research subject in the following cases: 1) when it is practically impossible to obtain informed consent in the course of the research process, 2) there is no reason to presume the research subject's refusal to consent, 3) even if consent is waived, the risk to the research subject is extremely low. Even in this case, it is limited to a retrospective study in which patients’ information is anonymized.

**Text changes for reviewer’s comment #4**

**Materials and Methods section, Patient enrollment paragraph**

Protocols of this study were approved by our Institutional Review Board (IRB No.: 2019-02-009), and the requirement to obtain informed consent was waived in this retrospective analysis.

🡪

Protocols of this study were approved by our Institutional Review Board (IRB No.: 2019-02-009), and the requirement to obtain informed consent was waived because this study is a retrospective study in which patients’ information is anonymized and this study might provide valuable knowledge to improve the patient care.
